# The many roads to mitochondrial dysfunction in neuroimmune and neuropsychiatric disorders

**DOI:** 10.1186/s12916-015-0310-y

**Published:** 2015-04-01

**Authors:** Gerwyn Morris, Michael Berk

**Affiliations:** 1 Tir Na Nog, Bryn Road seaside 87, Llanelli, Cardiff, Wales SA152LW UK; 2 grid.1021.20000000105267079IMPACT Strategic Research Centre, School of Medicine, Deakin University, PO Box 291, Geelong, 3220 Australia; 3 grid.431240.0Orygen Youth Health Research Centre and the Centre of Youth Mental Health, Poplar Road 35, Parkville, 3052 Australia; 4 grid.1008.9000000012179088XThe Florey Institute for Neuroscience and Mental Health, University of Melbourne, Kenneth Myer Building, Royal Parade 30, Parkville, 3052 Australia; 5 grid.1008.9000000012179088XDepartment of Psychiatry, University of Melbourne, Level 1 North, Main Block, Royal Melbourne Hospital, Parkville, 3052 Australia

**Keywords:** Autism, Bipolar disorder, Schizophrenia, Chronic fatigue syndrome, Cytokines, Depression, Immune dysfunction, Inflammatory, Mitochondrial dysfunction, Multiple sclerosis, Nitric oxide, Oxidative stress, Parkinson’s disease, Peroxynitrite, Psychiatry, Neurology

## Abstract

**Background:**

Mitochondrial dysfunction and defects in oxidative metabolism are a characteristic feature of many chronic illnesses not currently classified as mitochondrial diseases. Examples of such illnesses include bipolar disorder, multiple sclerosis, Parkinson’s disease, schizophrenia, depression, autism, and chronic fatigue syndrome.

**Discussion:**

While the majority of patients with multiple sclerosis appear to have widespread mitochondrial dysfunction and impaired ATP production, the findings in patients diagnosed with Parkinson’s disease, autism, depression, bipolar disorder schizophrenia and chronic fatigue syndrome are less consistent, likely reflecting the fact that these diagnoses do not represent a disease with a unitary pathogenesis and pathophysiology. However, investigations have revealed the presence of chronic oxidative stress to be an almost invariant finding in study cohorts of patients afforded each diagnosis. This state is characterized by elevated reactive oxygen and nitrogen species and/or reduced levels of glutathione, and goes hand in hand with chronic systemic inflammation with elevated levels of pro-inflammatory cytokines.

**Summary:**

This paper details mechanisms by which elevated levels of reactive oxygen and nitrogen species together with elevated pro-inflammatory cytokines could conspire to pave a major road to the development of mitochondrial dysfunction and impaired oxidative metabolism seen in many patients diagnosed with these disorders.

## Background

Syndromic or non-syndromic mitochondrial diseases, classified as cytopathies or encephalomyopathies, arise as a result of mutations in mitochondrial or nuclear DNA [1]. However, mitochondrial dysfunction and impaired bioenergetics are implicated in the pathogenesis of many chronic illnesses, mainly neuroimmune or autoimmune in nature, despite these not being currently categorized as primary mitochondrial diseases [1-5]. Mitochondrial dysfunction with concomitant oxidative stress is evidenced in the brains and periphery of many patients with the diagnoses of multiple sclerosis (MS) [6], chronic fatigue syndrome (CFS) [6], Parkinson’s disease (PD) [7], and autism [8].

Mitochondrial dysfunction in such individuals may well result from the presence of oxidative stress, as there is now ample evidence implicating oxidative stress as one of the major contributing factors in the development of mitochondrial dysfunction and compromised bioenergetic performance [9-13]. In fact, the causative role of chronic oxidative stress in the development of mitochondrial damage and localized or systemic bioenergetic failure has now been established beyond reasonable doubt [4,14-16]. Chronic oxidative stress develops in a cellular environment whenever production of reactive nitrogen species (RNS) and reactive oxygen species (ROS) exceeds the clearance ability of the cell’s antioxidant defenses such as the glutathione (GSH) and thioredoxin systems [17-19]. ROS and RNS are natural products of oxidative phosphorylation [18,20]. These reactive species can also be generated by activated inflammatory cells, including macrophages and microglia [21-24]. Oxidative stress and chronic inflammation are inextricably interconnected.

Oxidative stress activates a number of transcription factors, such as NF-kappaB and activated protein 1, leading to the production of pro-inflammatory cytokines (PICs), various chemokine species, and activation and proliferation of lymphocytes. The activation of other immune cells in turn leads to the production of more ROS and RNS, principally in the form of superoxide, nitric oxide (NO), and peroxynitrite [24-27]. The tissue damage characteristic of chronic inflammation is mediated directly by macrophages, neutrophils, and eosinophils via the production of PICs [28]. This intricate bidirectional self-amplifying and self-sustaining relationship between the development of chronic oxidative stress and chronic systemic inflammation is sometimes described as an ‘autotoxic loop’ [25,29].

ROS and RNS can also contribute to the development of chronic oxidative stress and inflammation via the oxidative and nitrosative modification of proteins, lipids, and DNA, resulting in modification of DNA bases and tertiary protein structure, lipid peroxidation of cell membranes, and the production of highly reactive aldehydes and ketones. The net result of these processes is the indirect and direct formation of damage-associated molecular patterns capable of activating pathogen sensing receptors on the surface and in the cytoplasm of immune cells [15,29-32].

The origin of oxidative stress in the brains of people suffering from a range of neuroimmune diseases, such as MS and PD, is still a matter of debate. There is now however strong evidence supporting the hypothesis that the oxidative stress in the brains of such patients stems from the transduction of inflammatory signals to the brain following the establishment of chronic inflammation and oxidative stress in the periphery. There is ample evidence demonstrating that systemic inflammation can lead to the development of chronic neuroinflammation [33-35]. Communication of inflammatory signals to the brain is mediated by PICs via a number of routes, including innervation of the vagus nerve, carrier-enabled transport across the blood brain barrier (BBB), activation of endothelial cells within the BBB and perivascular macrophages, and finally via transport through circumventricular organs devoid of a functional BBB [36,37]. The transduced inflammatory signals may lead to the development of chronic neuroinflammation via the activation of microglia if of sufficient intensity and/or duration or lead to the development of ‘primed’ microglia [34,36,38]. Microglial priming involves the up-regulation of a range of surface receptors such as MHC class II, CD11b, and CD11c integrins, co-stimulatory molecule CD86, and Toll-like receptor TLR4 [38].

Following the up-regulation of these receptors, such microglia become exquisitely sensitive to subsequent inflammatory stimuli, leading to an exaggerated production of neurotoxic molecules that may exacerbate the pre-existing pathology and may even accelerate the progression of existing neuroinflammatory or neurodegenerative diseases [39-41]. Activated microglia exert their neurotoxic effects by releasing PICs, such as tumor necrosis factor (TNF), interleukin (IL)-1, IL-6, and interferon (IFN), and free radicals including superoxide NO and peroxynitrite as well as inflammatory molecules such as prostaglandin E2. Moreover, TNF, IL-1, and IFN can act as secondary sources of RNS and other inflammatory molecules acting as potent inducers of inducible NO synthase (iNOS) and via their capacity to upregulate Cox-2 with the resultant production of prostaglandin E2 [36,38,42]. While the relationship between the establishment of chronic systemic inflammation and the development of chronic neuroinflammation is highlighted by numerous authors, the increase in levels of systemic inflammation following the development of neuroinflammation is perhaps under-appreciated. This may occur via a number of different mechanisms, including cytokine leakage from the central nervous system (CNS) into the circulation, increased cytokine synthesis in the periphery, primarily in the liver, and the escape of antigenic molecules likely activating Toll-like receptors on peripheral immune cells flowing ingestion by antigen presentation cells [38,39,43-45]. While these mechanisms could account for the presence of activated cell-mediated immunity in patients with MS, CFS, PD, bipolar disorer, depression, schizophrenia and autism as discussed below, they would not appear to explain the changes in lymphocyte populations and T cell differentiation patterns seen in illnesses like MS and PD. Whatever the cause, chronic inflammation in the body and/or the brain is characterized by the presence of elevated ROS and RNS together with increased levels of PICs.

The aim of this paper is to outline how excessive levels of PICs, notably TNF-α, ROS, and RNS, can lead to mitochondrial dysfunction and compromised bioenergetics in PD, MS, CFS, depression, bipolar disorder, schizophrenia and autism, by inhibiting the electron transport chain (ETC), the tricarboxylic acid cycle, and fatty acid oxidation, adversely affecting the activity and structure of structural and regulatory proteins and the integrity of essential functional lipid membranes. In short, this paper aims to demonstrate that chronically elevated levels of these pro-inflammatory entities are common denominators in paving the many roads to mitochondrial dysfunction.

## Discussion

### Immune dysfunction, oxidative stress, and mitochondrial dysfunction in MS

#### Evidence of immune dysfunction in MS

Chronic activation of the humoral and innate arms of the immune system in both the periphery and the CNS is a characteristic finding in MS patients. Commonly reported abnormalities include elevated levels of activated T helper (Th)2, Th17, and Th1 T cells, abnormal function of regulatory T cells, and activated naive B cells with impaired tolerance together with changes in the overall B cell subpopulation distribution pattern [6,29,46]. The relation between an activated peripheral immune system and the development of neuro-inflammation is thrown into stark relief in relapsing remitting MS by the proven efficacy of treatment with the monoclonal antibodies rituximab [47] and natalizumab [48], which target peripheral immune cells and ameliorate disease activity in the CNS [49]. Increased TNF-α levels in the periphery often precede active disease, and levels of this cytokine predict disability levels as measured by the expanded disability status scale (EDSS) [50-52]. Significant increases in plasma levels of IL-2, IL-1β, IL-4, and IL-13 have also been reported [53]. Given the strong positive relationship between TNF-α levels and degree of physical disability, it is of theoretical relevance that the levels of TNF-α and other PICs correlate significantly with the severity of fatigue which affects the vast majority of people with this illness [6,54-56]. The existence of a chronically activated peripheral immune system goes some way to explain the development and/or the maintenance of chronic systemic inflammation seen in sufferers of this disease, which we will now turn to illustrate. However, before doing so, this would seem to be an opportune juncture to emphasize the accumulating and persuasive evidence that a diagnosis of MS represents a spectrum of illnesses where different disease processes converge to produce a similar pathology [57,58]. For a fuller consideration of the evidence that has led many workers to this conclusion, the reader is referred to the work of Ortiz et al. [29].

#### Evidence of chronic oxidative stress in MS

There is now ample evidence highlighting the pivotal role of oxidative stress in the pathogenesis of MS [59-61]. Several authors have reported the existence of oxidative damage in the brain cerebrospinal fluid (CSF) and blood of MS sufferers [29,62]. Elevated levels of protein carbonyls have been detected in post-mortem brains of patients suffering from this disease [63]. Significantly elevated levels of other surrogate markers of oxidative stress have also been detected in the CSF and plasma of MS patients [60,63,64]. Studies investigating markers of oxidative and nitrosative stress in CSF have demonstrated increased levels of ethane and pentane, which are acknowledged markers of lipid peroxidation [65], malondialdehyde [66], hydroxynonenal [29], and isoprostanes [67]. Nitrotyrosine, a surrogate marker for peroxynitrite formation, is often found in active demyelinated lesions [68]. Unsurprisingly, iNOS levels are also elevated in lesions [69] and in CSF of patients with this illness [66]. High levels of NO, peroxynitrite, and superoxide have also been observed in spinal fluid extracted from patients with MS [66]. Interestingly, CSF levels of NO metabolites correlate positively with relapses [70]. Furthermore, Tasset et al. [9,71] have reported significant peripheral levels of oxidative stress in patients with relapsing remitting MS. Further evidence of the causative role of oxidative stress in the pathophysiology of MS is provided by a recent longitudinal study demonstrating that levels of oxidative stress increased dramatically during relapses but its presence was barely detectable in patients during remission [72]. It is also worthy to note that research teams have discovered that levels of oxidative stress in the blood and CSF correlate significantly and positively with levels of disability as measured by the EDSS [11,73]. The latter study also reported that levels of oxidative stress correlated significantly and positively with the extent of gadolinium-enhanced lesions [73].

#### Evidence of mitochondrial dysfunction in MS

Although the weight of evidence demonstrates that, while the development of pathology in the early stages of MS is largely driven by inflammation [74], mitochondrial dysfunction appears to have a crucial role in the progression of this disease [75,76]. Mitochondrial abnormalities in MS include altered structure and distribution coupled with a wide array of molecular and biochemical abnormalities [18,75,77-79]. Oxidative damage to mitochondrial DNA and impaired Complex I activity is a characteristic finding in active MS lesions [80]. Complex I and Complex III activity is also reduced in normal tissue within the motor cortex [81]. Complex IV activity is also decreased in lesions as well as in normal-appearing white and grey matter [82,83]. Studies utilizing NMR spectroscopy have demonstrated evidence of globally impaired bioenergetics and increased production of lactate in the CSF [84,85]. Lazzarino et al. [86] provided tantalizing evidence in a longitudinal investigation suggesting a global impairment of adenosine triphosphate (ATP) synthesis in MS when they reported that progressive central ATP depletion over a 3-year period correlated significantly and positively with increased physical disability as measured by changes in EDSS.

### Immune dysfunction, oxidative stress, and mitochondrial dysfunction in autism

#### Immune abnormalities in autism

A diagnosis of autism similarly in all probability represents a group of illnesses with heterogeneous etiology [87,88]. Epigenetics, rather than genetics, seemingly plays a dominant role in driving the development and persistence of these illnesses [89-91]. Several studies have investigated the presence of immune abnormalities in children afforded a diagnosis of autism herein described as children with autism (CWA) and in the parents of such children. Overall, the results demonstrate that CWA and their immediate family members, especially mothers, display markers of autoimmunity, abnormal cellular immunity, and aberrant expression of cytokines and other soluble mediators [92-95]. Abnormal expression of PICs and anti-inflammatory cytokines and their signaling effector molecules is commonly detected in CWA. These findings have been noted in the brain [96-99], gastrointestinal tract [100,101], and peripheral blood [102,103]. CWA commonly have increased plasma IL-1Β and abnormal cellular IL-1Β responses following mitotic stimulation of peripheral mononuclear blood leucocytes [103,104]. Abnormal levels of IL-6 in peripheral blood [103,105] and the brain [92,96,106] is another common finding. The observations relating to elevated levels of PICs extend to TNF-α [107,108] and INF-γ, which are once again elevated in the brain [92] and the peripheral circulation, interestingly, correlated with the levels of other inflammatory mediators such as NO [109,110]. The vast range of immune abnormalities displayed by many CWA is beyond the scope of this paper. An interested reader is referred to previous work for a more in depth treatment of the issue [94,103]. What remains unclear is if this evidence of immune dysregulation reflects a narrow sense of immunity in terms of cellular defense against exogenous pathogens or reflects dysregulation of signaling moieties with a wider range of intracellular signaling roles.

#### Evidence of oxidative stress in autism

The presence of chronic oxidative stress is commonly reported in CWA [111-114]. Interestingly, this abnormal state is sometimes reported in parents [115]. Several authors have also reported genetic abnormalities in GSH pathways in CWA [116-119], and some of these abnormalities correlate positively with the severity of symptoms [2,120]. Several researchers have detected lower concentrations of reduced GSH increased concentrations of oxidized GSH and a decreased GSH/glutathione disulfide ratio [117,121,122]. The mitochondrial reduced GSH reserve appears decreased in at least some children afforded this diagnosis compared to healthy controls. Additionally, in many studies, decreased GSH levels and several other markers of increased oxidative stress correlate positively with disease severity [123,124]. Other authors have reported a positive correlation between the severity of gastrointestinal dysfunction and surrogate markers of oxidative stress [125]. It is worthy of note that the aforementioned studies measured markers of oxidative stress in the periphery, but there is also a robust body of evidence demonstrating the existence oxidative stress in post-mortem brain samples from CWA compared to healthy controls [126-132].

#### Mitochondrial dysfunction and impaired bioenergetics in autism

Numerous workers have also reported the presence of mitochondrial dysfunction in CWA [133-139]. In many instances, biomarkers of mitochondrial dysfunction appear associated with disease severity [140,141]. It must be stressed, however, that not all children afforded a diagnosis of autism display evidence of mitochondrial dysfunction, as might be expected if this diagnosis actually represents several different diseases. Systematic reviews place the percentage of CWA displaying evidence of mitochondrial dysfunction as between 30 and 50% [114,142]. Historically, the bulk of published literature examining bioenergetic impairments have focused on blood and urine measures. However, an increasing number of researchers in recent years have reported evidence of impaired mitochondrial function in the brains of CWA compared to healthy controls [126,129,132,143-146]. Studies involving ^31^P-magnetic resonance spectroscopy have reported decreased production of ATP elevated levels of lactate, reduced levels of carnitine [140,147-149], and other measures of mitochondrial dysfunction [150,151].

### Immune dysfunction, oxidative stress, and mitochondrial dysfunction in Parkinson’s disease (PD)

#### Immune abnormalities in PD

There is evidence that a diagnosis of PD also represents a range of illnesses of heterogeneous etiology [152,153]. A wide array of peripheral immune abnormalities have been detected in patients with PD. The reduction in lymphocyte numbers in general and CD19 B, together with CD3 and CD4 subsets, is especially commonplace. Of the remaining subsets, an increased frequency of T cells secreting Th1 cytokines and a reduced frequency of Th2 cytokine-secreting T lymphocytes is also a common finding. For details of these immune abnormalities and the evidence supporting their existence, the reader is referred to an excellent review by Mosely et al. [45]. The cause of these immune abnormalities in the periphery is far from clear. One suggestion proposes that various elements of the adaptive and innate immune system could become activated as a result of the escape of CNS proteins into the periphery, which could function as damage-associated molecular patterns [30]. It is worthy of note that corrupted species of proteins specific to this disease, including the phosphorylated form of α-synuclein, are found in peripheral tissues in PD patients [154]. Other authors have suggested prolonged pathogen infection or chronic exposure to environmental toxins as the root cause of immune dysregulation and chronic inflammation seen in people with this illness [38]. Further, albeit indirect, evidence of abnormalities in immune and inflammatory pathways in patients with PD stems from the existence of elevated levels of Cox-2 and members of the NF-kappaB family in the *substantia nigra*, and elevated levels of IL-15, IL-10, and RANTES in the CNS and peripheral circulation in people afforded this diagnosis [155,156]. A number of authors have reported abnormally elevated serum levels of TNF-α and TNF receptor 1 in patients with PD [43,157,158]. Elevated levels of IL-6 have also been detected in the plasma of PD patients, which correlate positively with an increased disease risk [159]. Elevated levels of IL-1β has also been detected in the CSF and the strata of patients with PD, with the latter findings being post-mortem [160-162]. Abnormally, high levels of TNF-α and INF-γ are also commonly observed in the CSF and post-mortem tissue of people suffering from this illness [161,163]. It is also particularly noteworthy that peripheral immune responses have the capacity to trigger exacerbation of PD symptoms, probably on the basis of neuroinflammation [163-165].

#### Evidence of oxidative stress in PD

Oxidative stress is considered to be the common underlying mechanism driving cellular dysfunction and ultimate demise in genetic and idiopathic cases of PD. The wealth of evidence supporting this viewpoint includes increased levels of oxidized lipids [166], proteins and DNA [167], and decreased levels of reduced GSH in the *substantia nigra* of PD patients [168-171]. Other abnormalities indicative of oxidative stress observed in the *substantia nigra* and other regions of the brain include carbonyl modifications of soluble proteins [172,173], oxidized DNA [167,174], and increased levels of malondialdehyde and 4-hydroxy-2-nonenal, and reduced levels of polyunsatrurated fatty acids [175,176]. Nitration and nitrosylation of several proteins, including of alpha-synuclein and parkin, have also been repeatedly documented [177-179]. Many studies have also reported strong evidence of chronic oxidative stress in PD blood and CSF strongly suggesting that PD is a generalized disease [167,180-185].

#### Mitochondrial dysfunction and bioenergetic abnormalities in PD

Early evidence demonstrating a link between mitochondrial dysfunction and the pathogenesis of PD involved a number of reports illustrating Complex I impairment in the post-mortem *substantia nigra* pars compacta of patients [186,187]. This Complex I deficiency is also evident in the frontal cortex of PD [188], and remarkably in peripheral tissues such as skeletal muscle [189] and platelets [190], strongly suggesting the presence of global impairment in mitochondrial Complex I activity in this disease. It is also worthy of note that oxidative damage to Complex I and subsequent complex miss-assembly is a common feature of isolated mitochondria in the brains of PD sufferers [191].

Decreased function of Complex III is also commonly detected in the platelets and lymphocytes of PD patients [190,192]. A strong link between impairments in the assembly of mitochondrial Complex III and an increase in free radical damage in the mitochondria isolated from PD patients has also been reported [193]. It is possible that the increase in free radical damage stems from an increased production in ROS and RNS. This increase in free radical release may be due to the increased leakage of electrons from Complex III (as explained below). Alternatively, the inhibition of Complex III assembly causes a severe reduction in the levels of functional Complex I in mitochondria [194], which could lead to an increase in free radical production through Complex I deficiency. The use of magnetic resonance spectroscopy has revealed evidence of *in vivo* widespread mitochondrial dysfunction in virtually every region of the brain in PD patients, demonstrating that bioenergetic abnormalities and a shift to anaerobic metabolism are not confined to the substantia nigra [195-197]. It is worth stressing, however, that studies investigating mitochondrial dysfunction in PD highlight that its pathophysiological heterogeneity as mitochondrial function is normal in many patients afforded this diagnosis [198].

### Immune dysfunction, oxidative stress, and mitochondrial dysfunction in chronic fatigue syndrome (CFS)

#### Immune abnormalities in patients with CFS

Metzger et al. [199] reported evidence of abnormal Th17 T cell activity in driving the symptoms of people within their trial cohort. It is of interest that Th17 cells have a critical role in mucosal defense, with particular functions in gut and respiratory defenses. Other studies examining receptors expressed on the surface of T cells extracted from people with CFS have also provided evidence of impaired T cell activation with a possible Th17 differentiation pattern [200,201]. Other studies report the presence of activated but anergic T cells (Review [6]). Recent evidence has challenged the view that people with CFS display immune abnormalities consistent with a Th2 pattern of T cell differentiation. While some patients present with a Th2 profile and a preponderance of anti-inflammatory cytokine production, others present with a Th1 or possibly Th17 profile, with the synthesis of PICs being dominant [202-204]. Elevated levels of TNF-α and IL-1β are, in fact, particularly commonplace observations in patients recruited into studies using the internationally agreed diagnostic guidelines [202,205-211]. However, some patients also present with elevated levels of Foxp3-expressing regulatory T cells likely in an attempt to counter the proliferation of activated T cells [212,206]. While there is ample evidence that many patients afforded a diagnosis of CFS display profound immunological abnormalities characteristic of a chronically activated but dysregulated peripheral immune system, it must be stressed that some patients with such a diagnosis do not (review [213]). Such disparate often conflicting findings, between and within cohorts, are typical of studies investigating the existence of diverse neuropathology (review [1]). These and other lines of evidence strongly argue that a diagnosis of CFS does not represent a unitary illness with a single pathogenesis and pathophysiology but rather represents a spectrum of illnesses where different pathophysiological processes converge to produce a very similar phenotype [214-217]. This is a core issue across neurobiology, where diagnoses, in the absence of coherent knowledge of pathophysiology, are made on the basis of symptomatology. Nowhere in the rest of medicine does phenomenology parallel pathophysiology, nor should we expect it to do so in neuropsychiatric disorders. The situation is thus made more complex as a diagnosis of CFS is also afforded to people who present with weariness of uncertain or overtly psychological origin either with or without additional non-specific and intermittent symptoms [218-220]. Furthermore, patients afforded a diagnosis of CFS using one of these localized or department-specific protocols are often recruited into studies using predetermined scores on varius non-specific fatigue scales and symptom inventories [218,221]. It must be emphasized that there is therefore no evidence of a consistent pattern immune or neurological abnormalities and, indeed, no evidence of mitochondrial dysfunction in patients afforded a diagnosis of CFS using any of these alternative approaches [213,222-224].

#### Evidence of chronic oxidative stress in patients with a diagnosis of CFS

Elevated oxidative stress is an almost invariant finding in studies investigating this phenomenon in patients afforded a diagnosis of CFS, with many studies reporting a significant positive correlation between markers of oxidative stress and symptom severity. Several authors have reported that oxidative and nitrosative stress measures demonstrate a significant and positive correlation with symptom severity [225-232]. Miwa and Fujita [233] reported that a fall in the oxidative stress levels of patients corresponded with their transition into remission. Several authors have reported systemic increases in markers of nitrosative and oxidative stress including malondialdehyde, isoprostane, 8-OH-deoxyguanosine, 2,3-diphosphoglyceric acid, thiobutyric acid, and protein carbonyls [225-230,233-235]. iNOS and NO production is significantly increased in many patients relative to levels in normal controls [225,236]. Oxidative imbalance is reported in skeletal muscle, and its severity has been reported to correlate positively with objective measures of muscle fatigability reported by affected patients [237]. Finally, a recent NMR spectroscopy study reported significantly decreased cortical GSH levels in the brains of patients diagnosed according to the Fukuda guidelines [232]. As this review has emphasized, oxidative stress and chronic inflammation, metaphorically like pyrexia, are ubiquitous findings in diverse and seemingly unrelated disorders.

#### Evidence of mitochondrial dysfunction in patients afforded a diagnosis of CFS

A number of studies investigating bioenergetic performance in patients diagnosed with CFSs have reported evidence of mitochondrial dysfunction, including a loss of mitochondrial membrane integrity and oxidative damage to translocatory proteins in a class of peripheral mononuclear blood cells [238-240]. These findings support earlier work reporting abnormal mitochondrial morphology in muscle biopsy tissue and defects in aerobic metabolism not characteristic of muscle disuse [241-244]. Several authors utilizing ^31^P NMR spectroscopy to investigate the bioenergetic performance of striated muscles in CFS sufferers have reported profound defects in oxidative phosphorylation, as evidenced by direct or surrogate markers of ATP re-synthesis and low basal levels of ATP production [245-250]. Another line of evidence, once again produced via the use of NMR spectroscopy, demonstrates the existence of abnormal lactate responses to exercise in some patients with CFS [251-253]. Notably, the observed changes in the heart rate of patients coupled with an examination of muscle fiber morphology could not be attributed to deconditioning [252,253]. The pathophysiological heterogeneity within the trial cohorts, however, was striking, with approximately 50% of patients displaying these abnormalities while the other cohort members displayed no metabolic abnormalities in muscle function [251-253]. This gives further support to the view that a diagnosis of CFS, even using internationally agreed criteria as in these studies, does not represent a single illness. This is even more graphically illustrated in a study by Barnes et al. [254], where only 20% of patients were found to have muscle defects in oxidative metabolism. In a recent review, Filler et al. [223] concluded that there was ample evidence of mitochondrial dysfunction and impaired bioenergetic performance in patients afforded a diagnosis of CFS, but once again it was confined to patients diagnosed according to internationally agreed criteria and not apparent in all patients. Vermeulen et al. [255] conducted two exercise tests using cycle ergonometry, on CFS patients on consecutive days, and found that patients attained their anaerobic threshold at a markedly lower oxygen consumption than their putatively healthy counterparts in the first test. Importantly, the anaerobic threshold attained by patients occurred at a much lower oxygen consumption in the subsequent test. These findings were also evidenced in the patients maximal exercise capacity relative to healthy controls, which was also attained at a much lower oxygen capacity than the control group and correlated with differences in ATP production [255]. In a follow-up study, Vermeulen and Vermeulen [256] examined exercise performance in a cohort of CFS patients and reported a loss in the linear relationship between heart rate and cardiac output and the dissipation of oxygen concentration gradient between venous and arterial blood characteristic of mitochondrial dysfunction. Finally, the use of NMR spectroscopy also revealed that some patients display significantly increased ventricular lactate levels, indicative of widespread mitochondrial dysfunction [232,257]. Readers interested in a detailed explanation of the characteristic changes in exercise physiology characteristic of mitochondrial dysfunction are referred to previous studies [1,258]. Again, as with oxidative stress, these increases in lactate are found in seemingly divergent disorders that are not overtly mitochondrial in nature, including schizophrenia [259], and reflect a shift to anaerobic or possibly aerobic glycolysis as a mode of ATP generation.

### Immune dysfunction, oxidative stress, and mitochondrial dysfunction in bipolar disorder

#### Evidence of immune dysfunction in bipolar disorder

Many lines of evidence converge to suggest a role of immune dysregulation in bipolar disorder. Bipolar disorder is commonly associated with autoimmune disorders including MS, thyrotoxicosis, ulcerative colitis, psoriasis, and rheumatoid arthritis [260]. To date, a number of studies have consistently shown that there are elevated C-reactive protein levels in bipolar disorder, both in acute mania and remission [261,262]. Similarly, TNF-α and IL-6 have shown consistent patterns of elevation in the disorder. Interestingly, there is a suggestion of stage-specific changes in these markers, with elevated levels of PICs in early and late stages, but loss of elevated IL-10, an anti-inflammatory cytokine, in late stage illness [263,264]. A recent meta-analysis showed that there are higher concentrations of TNF-α, soluble IL-2 receptor, and soluble TNF receptor type 1 in bipolar patients than in controls [265]. The study did not find significant differences between bipolar disorder patients and healthy control subjects for IL-1, IL-2, IL-5, IL-6, IL-8, IL-10, IL-12, IL-1β, IL-1 receptor antagonist, IFN-γ, transforming growth factor-β1 (TGF-β1), and TNF receptor type 2 [265]. A range of anti-inflammatory agents, including aspirin, minocycline, N-acetylcysteine, curcumin, anti-TNF-α agents, celecoxib, and omega-3 fatty acids are being investigated as an adjunct to treatment as usual for use in mood disorders, and the extant, albeit preliminary, evidence shows promise [266]. Finally, many of the known risk factors for the development of mood disorders drive systemic inflammation, including physical inactivity, stress, poor diet, obesity, smoking, atopy, altered gut permeability, dental caries, vitamin D deficiency, and dysregulated sleep [35].

#### Evidence of chronic oxidative stress in patients with a diagnosis of bipolar disorder

There is consistent evidence from peripheral marker studies that the brain’s primary antioxidants, GSH, catalase (CAT), superoxide dismutase (SOD), and GSH peroxidase are altered in those with bipolar disorder [267]. In addition, there is post-mortem data that GSH is depleted in those individuals who have bipolar disorder, as well as in people with schizophrenia [268]. In parallel, there is now meta-analysis level data showing increased markers of oxidative stress. The most consistent findings are increased lipid peroxidation, DNA/RNA damage, and raised NO in bipolar disorder compared to controls, with high effect sizes for lipid peroxidation [269]. Oxidative damage to proteins (protein carbonylation) is also consistently shown [270]. Clinical data suggests there are correlations between illness severity and the extent of oxidative stress, such that those with greater illness duration, and a larger number of prior episodes show decreased antioxidant defenses [264,271]. Atypical antipsychotic drugs, as a class, possess redox-active properties although the extent to which they mediate their pharmacological benefits is uncertain [272,273]. Lithium and valproate also have extensive effects on oxidative markers [274,275]. Remission in bipolar depression was mirrored by increases in oxidative defenses and reductions in oxidative stress measures [276]. Lastly, many of the known environmental precipitants and risk factors for depression appear to be transduced via redox signaling [277].

#### Mitochondrial dysfunction and bioenergetic abnormalities in bipolar disorder

Of all the disorders mentioned in this review, bipolar disorder has the highest face validity as a primary disorder or mitochondrial bioenergetics, being a biphasic disorder of symptomatically increased and decreased energy and activity. Mania is known to be associated with increased brain energy generation, while depression is associated with decreased energy generation [278]. Patients with bipolar disorder have a higher prevalence of primary mitochondrial disorders than the general population, particularly mitochondrial encephalomyopathy, lactic acidosis, and stroke-like episodes [279]. Abnormalities in brain and lymphocyte mitochondrial distribution and morphology using electron microscopy have been observed in bipolar disorder [280]. Proton magnetic resonance imaging studies show an increased brain glutamate/glutamine ratio in bipolar disorder [281]. This increased excitatory glutamate creates a high energy demand. Additionally, elevated lactate levels and decreased intracellular pH suggest a shift to glycolysis and imply dysregulation of mitochondrial bioenergetics [282,283]. Furthermore, there is evidence of changes in expression of genes encoding for mitochondrial complexes, particularly Complex I and IV [284,285]. Mitochondrial dysfunction is thus a target of novel therapeutic endeavors in this disorder [286].

### Immune dysfunction, oxidative stress, and mitochondrial dysfunction in major depression (MDD)

#### Evidence of immune dysfunction in MDD

MDD is characterized by evidence of activated cell-mediated immunity with many patients demonstrating (Th1) style response with elevated levels of IFN-γ [287,288]. Several meta-analyses and numerous recent studies have demonstrated elevated levels of IL-1β, TNF-α, and IL-6 together with increased levels of neopterin and soluble IL-2 receptors, which globally indicate increased cell-mediated immunity and macrophage activity [289-293]. However, there is evidence of biologically distinct MDD subtypes where Th2 cytokines predominate [294]. Investigating therapeutic responses to the antidepressant duloxetine, the existence of patients with a Th2-biased cytokine profile whose positive response to treatment was indicated by a Th1 shift in their cytokine profile was reported; this contrasted with other patients whose baseline cytokine profile was characteristic of a Th1 profile which shifted towards a Th2 cytokine pattern in response to treatment [294]. This supports very similar findings in an earlier study by the same authors [295] and is in line with the work of other researchers where the positive effects of treatment were evidenced by increased levels of TNF-α and decreased levels of IL-4 [296]. Other findings include evidence of increased numbers of circulating Th17 T cells, diminished numbers of regulatory T cells (Tregs), and a significantly increased Th17/Treg ratio [297].

#### Evidence of oxidative stress in patients with a diagnosis of MDD

Nitrosative and oxidative stress are now considered to play a major role in the pathophysiology of MDD [298-301]. It seems likely that this state arises as a result of elevated production of ROS and RNS and compromised cellular antioxidant defenses. Galecki et al. [302] reported elevated levels of SOD and CAT activity and a global deficit of antioxidant defenses [302]. Deficiencies have also been reported in other antioxidant compounds such as vitamins C and E [303-305]. There is also evidence of widespread lipid peroxidation in crucial areas of the brain such as the prefrontal cortex whose levels in female patients correlate with the severity of symptoms [306,307]. The existence of lipid peroxidation is further evidenced by high levels of serum malondialdehyde and oxidative damage to lipids in peripheral tissues [302,308]. Elevated levels of urine and plasma isoprostane and 8-oxo-2’-deoxyguanosine have also been reported, which bears testimony to excessive levels of ROS and RNS produced outside the brain [303-305]. It is significant that the concentration of oxidative stress markers in the periphery correlates positively with the chronicity and severity of the illness irrespective of patient gender [299,307-309]. Given such a relationship, it is not surprising that antioxidant compounds are being trialed as potential antidepressant treatments [310,311]. The level of oxidative damage to lipids and DNA is sufficient to form neoepitopes and provoke antibody responses [312,313]. The presence of oxidative damage to mtDNA bears further testimony to the severity of oxidative stress in sufferers of this illness [314,315].

#### Evidence of bioenergetic impairments and mitochondrial dysfunction in patients with MDD

As previously discussed, there is copious evidence implicating the activation of immuno-inflammatory pathways and chronic oxidative and nitrosative stress in the genesis, persistence, and severity of MDD [316-320]. However, there is a growing awareness that the archetypal symptoms of MDD, such as neurocognitive impairment, sleep disturbances lethargy, fatigue, and loss of motivation, may also be driven by mitochondrial dysfunction primarily in the domain of the ETC [321-323]. This is perhaps expected as the bidirectional association between elevated PICs, chronic oxidative and nitrosative stress, and mitochondrial dysfunction has been clearly established and will be discussed in detail below [1,36]. There is also accumulating evidence that mitochondrial dysfunction plays a role in the etiology of the illness.

Several neuroimaging studies utilizing PET or SPECT technology have detected impaired bioenergetic metabolism in numerous regions of MDD patient brains, notably in the basal ganglia and prefrontal cortices [6,321]. Other authors have reported widespread abnormalities in blood flow, energy and glucose metabolism, as well as a reliance on glycolysis as a source of ATP production [324-327,321]. A number of studies have demonstrated the existence of mitochondrial dysfunction in the peripheral tissues of MDD patients, which is of interest, as it would be expected in light of growing data implicating systemic inflammation in the genesis of the illness [317]. For example, Gardner et al. [328] demonstrated that striated muscle mitochondria of patients with MDD, who concomitantly presented with physical symptoms, synthesized a significantly lower amount of ATP and showed impairments in respiratory chain enzyme activity, particularly at Complex III and IV [328]. Hroudová et al. [329] were the first research team to demonstrate mitochondrial dysfunction in the peripheral mononuclear blood cells of MDD patients, and this finding has been confirmed in an even more recent study by Karabatsiakis et al. [330]. These authors assessed mitochondrial respiration in intact peripheral blood mononuclear cells via the use of high-resolution respirometry using a healthy volunteer control group [330]. They demonstrated that MDD patients displayed grossly impaired mitochondrial functioning along several dimensions. Importantly, mitochondrial respiration correlated significantly and negatively with the severity of many depressive symptoms, notably loss of energy, fatigue, and difficulties concentrating strongly, suggesting a causative role for ATP shortage in the genesis of such symptoms [330].

### Immune dysfunction, oxidative stress, and mitochondrial dysfunction in schizophrenia

#### Evidence of immune dysfunction in schizophrenia

A range of immunological abnormalities have been detected by several authors in patients with schizophrenia [331,332]. Most researchers have focused on levels of plasma cytokines following mitogenic stimulation of peripheral blood mononuclear cells, and have broadly revealed the existence of a Th2-biased immune system, although the detailed picture is somewhat mixed [333-336]. However, more recent data also indicates the presence of elevated levels of TNF-α, IL-1β, and IL-6 in treatment-naive patients, which appear to drive the genesis and maintenance of neuroinflammation in at least some patients [337-340]. Moreover, accumulating data also indicates the existence of elevated numbers of effector and memory Th17 cells which are responsible for the development of neuropathology and autoimmunity in other illnesses [337]. The probable contribution of Th17 T cells to the pathophysiology of schizophrenia was highlighted in a recent study by Ding et al. [338]. These authors reported the presence of activated Th17 T cells in drug-naive first episode schizophrenia patients and also noted a significant positive relationship between the proportion of activated Th17 cells and the levels of IL-17, TNF-α, IL-6, and INF-γ with the negative symptom on the positive and negative syndrome scale [338]. Perhaps even more importantly, the proportion of Th17 cells decreased in patients displaying a positive response to risperidone which correlated positively with the change in score [338].

#### Evidence of oxidative stress in schizophrenia

Chronic systemic inflammation and oxidative stress is an invariant feature of schizophrenia [337,341,342]. It is now recognized that levels of inflammation and oxidative stress correlate with the level of cognitive impairment in patients with first episode schizophrenia [343]. It would also appear that levels of oxidative stress correlate with severity of positive symptoms [344]. It probably unsurprising to learn that oxidative and nitrosative stress is causatively implicated in the pathogenesis and pathophysiology of the illness [345-347]. Numerous authors have reported the presence of oxidative damage to proteins, lipids, and DNA [346,348]. There is evidence of ROS and RNS overproduction and reduced levels of antioxidants [349,350]. Numerous research teams have detected the presence of oxidative stress in the prefrontal cortex and CSF *in vivo* [351]. Post-mortem studies have revealed the presence of this phenomenon in the anterior cingulate cortex [352].

The presence of oxidative stress is not confined to the brains of those with schizophrenia, but is also found in plasma and peripheral tissues of patients [353-355]. Specific abnormalities include elevated levels of malondialdehyde and NO coupled with significantly reduced levels of GSH relative to healthy controls [344,356,357]. It is worthy of note that the presence of thiobarbituric acid reactive substances and protein carbonyls are seen both in the early and late stages of the disease [358]. It is also worthy of note that the presence and levels of molecules signifying the presence of oxidative and nitrosative stress correlate with the enzymatic activity of Complex I [359]. It is fair to state, however, that some controversy remains regarding the activities and levels of enzymatic antioxidant activity evaluated by SOD, CAT, and GSH peroxidase, with normal and abnormal levels and activities being reported by different research teams [360-362]. Reflecting the classification of Davis [363], a recent meta-analysis suggested that total antioxidant status, red cell catalase, and plasma nitrite are potential state or acuity markers of the disorder, while others, including red cell SOD, are trait markers [364].

#### Evidence of bioenergetic impairments and mitochondrial dysfunction in schizophrenia

Several authors demonstrated a range of ultrastructural abnormalities in the mitochondria of patients suffering from schizophrenia, which may play a major role in its pathogenesis [365,366]. Many of these studies have relied on the examination of post-mortem brain tissue by electron microscopy and abnormalities reported have included denuded numbers, reduced density, and significant defects in cross-sectional profiles [367,368]. Enlarged mitochondria with disrupted cristae have been detected in astrocytes of patients with longstanding illness, and may be the source of the progressive astrocyte dysfunction seen in the illness [369]. Interestingly, ultrastructural abnormalities in these organelles are not confined to the brain, as impaired mitochondrial numbers and density are also evident in peripheral blood mononuclear cells [370,371]. Abnormalities in Complex I activity are also a frequently reported finding [372,373]. The pattern of these abnormalities in the brain is somewhat inconsistent, but dependent on the location and function of the tissue sampled [374-377]. A recent study conducted by Akarasu et al. [378] detected Complex I hyperactivity in peripheral blood from schizophrenia patients, which the authors proposed as a diagnostic marker for the illness. Increased activity of Complex I in peripheral tissues was reported in an earlier study [375]. The evidence demonstrating impaired activity of mitochondrial complex enzymes induced by conformational changes in the frontal or prefrontal cortex appears to be more consistent, however, with the decreased activity of Complex I, III, and IV [377,379-381].

Impaired bioenergetics and an increase in glycolytic ATP production secondary to mitochondrial dysfunction has been detected in the post-mortem brains of schizophrenia patients via the use of NMR spectroscopy [382-384]. The same technique has also revealed elevated levels of lactate and a decrease in levels of pyruvate dehydrogenase, indicative of mitochondrial dysfunction and a shift to energy production via glycolysis in the CSF of patients with this illness [385,386]. Several studies have reported a positive association between a range of defects in the mitochondrial genome and the development of schizophrenia [387,388]. There is also a growing body of evidence suggesting an increased maternal transmission of schizophrenia, which may indirectly indicate a potential role for mitochondrial inheritance in the etiology of the illness [389,390]. Numerous authors have demonstrated an association between certain mitochondrial haplotypes and disease risk, and the associations also extend to age of onset[390]. In a similar vein, functional single nucleotide polymorphisms in mitochondrial genes encoding for Complex I, ATP synthase, and Cox subunits also confer an increased risk of developing the illness [391-393]. Table 1 provides a summary of the various mitochondrial and bioenergetic abnormalities recorded in the illnesses discussed above.

**Table 1:** **Range of mitochondrial abnormalities seen in MS, CFS, PD, AUT, MDD, Schiz and BPD**

**Mitochondrial abnormality**	**MS**	**CFS**	**PD**	**AUT**	**MDD**	**Schiz**	**BPD**
Ultrastructural abnormalities in mitochondria	+	++	+	+	+	+++	+
Evidence to shift in energy production via glycolysis	+	++	++	++		++	++
Mitochondrial dysfunction in peripheral immune cells	+	++	++	++	++	++	+
Mitochondria DNA damage	+					++	
Damage to the electron transport chain	++		+++	+		++	+
High lactate in brain or cerebrospinal fluid	+	+	+	+	+	++	+
Bioenergetic impairment Skeletal muscle		+++			+		
Decreased mitochondrial membrane potential		+	+				

MS, Multiple sclerosis; CFS, Chronic fatigue syndrome; PD, Parkinson’s disease; AUT, Autism; MDD, Major depression; Schiz, Schizophrenia; BPD, Bipolar disorder.

### The role of NO peroxynitrite and proinflammatory cytokines in the genesis of mitochondrial dysfunction and impaired oxidative metabolism

#### Nitric oxide (NO)-mediated impairment of energy production

NO impacts on mitochondrial performance and levels of ATP production in a number of ways, notably, by playing a major role in governing oxygen delivery [394,395] and, crucially, by inhibiting the performance of the ETC. NO inhibits the activity of Complex IV via a number of mechanisms, not least by acting as a competitive antagonist for the binding of oxygen to the enzyme’s active site [396-398]. NO also inhibits electron transfer between cytochrome b and c, namely the electron transfer at Complex III [399,400], which directly leads to increased production of ROS [401]. Finally, NO inhibits NADH dehydrogenase activity and electron transfer at Complex I [399,402,403]. Although the reaction with Complex III is somewhat ponderous [404], the reaction between Complex I and Complex IV, otherwise known as cytochrome c oxidase (COX), is extremely rapid and generally reversible. It is worth noting at this juncture that inhibition of these components of the ETC can be a source of pathology other than by direct inhibition of ATP production. Both reactions produce a number of derivatives responsible for generating the nitrosative stress of mitochondrial origin observed in a number of different illnesses, mainly neuroimmune or autoimmune [397,401,405-407]. NO reacts with the iron and copper ions of the heme-CuB and sulfhydryl groups located at the active site of COX [408-411], while inhibition of Complex I stems from the S-nitrosylation of Cys39 prominent on the ND3 subunit [410,412,413].

The inhibition of Complex I and COX is normally reversible but is less so following prolonged excessive production of NO [402,413,414]. While the reaction of NO with both ETC complexes is extremely rapid compared to the reaction with Complex III, inhibition of Complex IV following exposure to NO occurs within seconds or milliseconds [415], while inhibition of Complex I may occur within minutes [413]. The onset of NO inhibition on Complex I is slow (minutes) [413], whereas on COX is very fast (milliseconds to seconds) [415,396]. As previously discussed, when excessively elevated levels of NO persists in the cellular environment, enabling prolonged nitrosylation, the inhibition of COX and Complex I may become virtually irreversible [416-419], leading to a substantial inhibition of the ETC, impaired oxidative phosphorylation, and a decreased synthesis of ATP [1,36,396,420]. This situation leads to the induction of glycolysis even in an environment of abundant oxygen in an attempt to compensate for loss of ATP [421,422]. This is very similar, in principle, to the Warburg effect [423], and the weight of evidence suggests that the efficiency of this compensatory mechanism varies with cell type and location [424,425]. Prolonged nitrosylation of COX, however, likely overcomes this compensatory mechanism leading to a situation of chronic ATP depletion [396]. NO also has a positive effect on net ATP production by playing a crucial role in mitochondrial biogenesis within skeletal muscle [384]. This positive contribution is considered to be mediated by its capacity to upregulate the transcription of peroxisome proliferator-activated receptor gamma coactivator-1 (PGC-1α) [426-428]. In an inflammatory environment, however, this stimulatory effect is countered by the presence of elevated levels of TNF-α, which reduces the expression of PGC-α, a key regulator of energy metabolism, via the upregulation of NF-kappaB and p38 MAPK kinases [132,429,430]. Moreover, inhibition of the ETC predisposes to the excessive production of superoxide anions, which react with NO to form the highly dangerous peroxynitrite [1,36,402]. This reactive species has the capacity to compromise virtually every element and system involved in the generation and regulation of energy production as we will now illustrate.

### Peroxynitrite-mediated impairment of energy production

Peroxynitrite has a much longer half-life than its molecular ancestors and is much more reactive [431,432]. Thiol oxidation and nitration of tyrosine residues are the major mechanism by which peroxynitrite induces conformational change in proteins [433,434]. Peroxynitrite also causes oxidative damage to mitochondrial structural proteins and enzymes and peroxidative damage to lipids within membranes leading to profound changes in function and membrane integrity [1,435,436]. Peroxynitrite inhibits mitochondrial respiration by inactivation of ETC I and III [437,438]. Inactivation of mitochondrial electron transport enzymes increases mitochondrial production of superoxide and hydrogen peroxide generated by mitochondria [437], creating adaptive and synergistic damage [439].

Peroxynitrite can make an indirect contribution towards mitochondrial dysfunction by inhibiting SOD [440] and glutaredoxin [441], and by oxidizing reduced GSH and other thiols [442-444]. GSH depletion, in turn, exacerbates peroxynitrite-induced pathology [445]. This intimate bidirectional relationship with oxidative and nitrosative stress is reviewed in Morris and Maes [1] and Morris et al. [36]. The oxidation of critical cysteine groups by this highly reactive species inactivates a plethora of enzymes playing indispensable roles in bioenergetic processes, including glyceraldehyde-3-phosphate dehydrogenase [446,447], NADH dehydrogenase [403], creatine kinase [448], succinate dehydrogenase [449], cytochrome c reductase [450], and ATP synthase [451,452]. These enzymes are also inactivated by nitration of tyrosine damage to their iron sulfur centres [451,452] and, thus, are highly prone to inactivation by chronically elevated levels of peroxynitrite. The redox activity of cytochrome C is severely impaired by nitration and, hence, this cytochrome is also very readily disabled in an environment of chronically elevated peroxynitrite. Nitration of cytochrome c significantly elevates its peroxidatic activity, leading to increased synthesis of hydrogen peroxide accelerating further oxidative corruption of mitochondrial proteins [453,435]. Peroxynitrite also disrupts the ferrous-sulfur active site of the tricarboxylic acid cycle enzyme aconitase, leading to its inhibition and impairing ATP production [454,455]. The enzyme nicotinamide nucleotide transhydrogenase, which catalyzes the reduction of NAD, is another crucial mitochondrial enzyme readily inactivated by peroxynitrite-mediated nitration and oxidation [456]. The subsequent depletion of NADPH impairs the ability of mitochondria to further regenerate reduced GSH, exacerbating the pre-existing oxidative stress within the organelle [457,458]. Chronically elevated levels of peroxynitrite lead to mitochondrial membrane depolarization [459,460], which is probably mediated by thiol oxidation of cysteine residues of proteins within the permeability transition pore complex [461,457].

**Figure 1:**
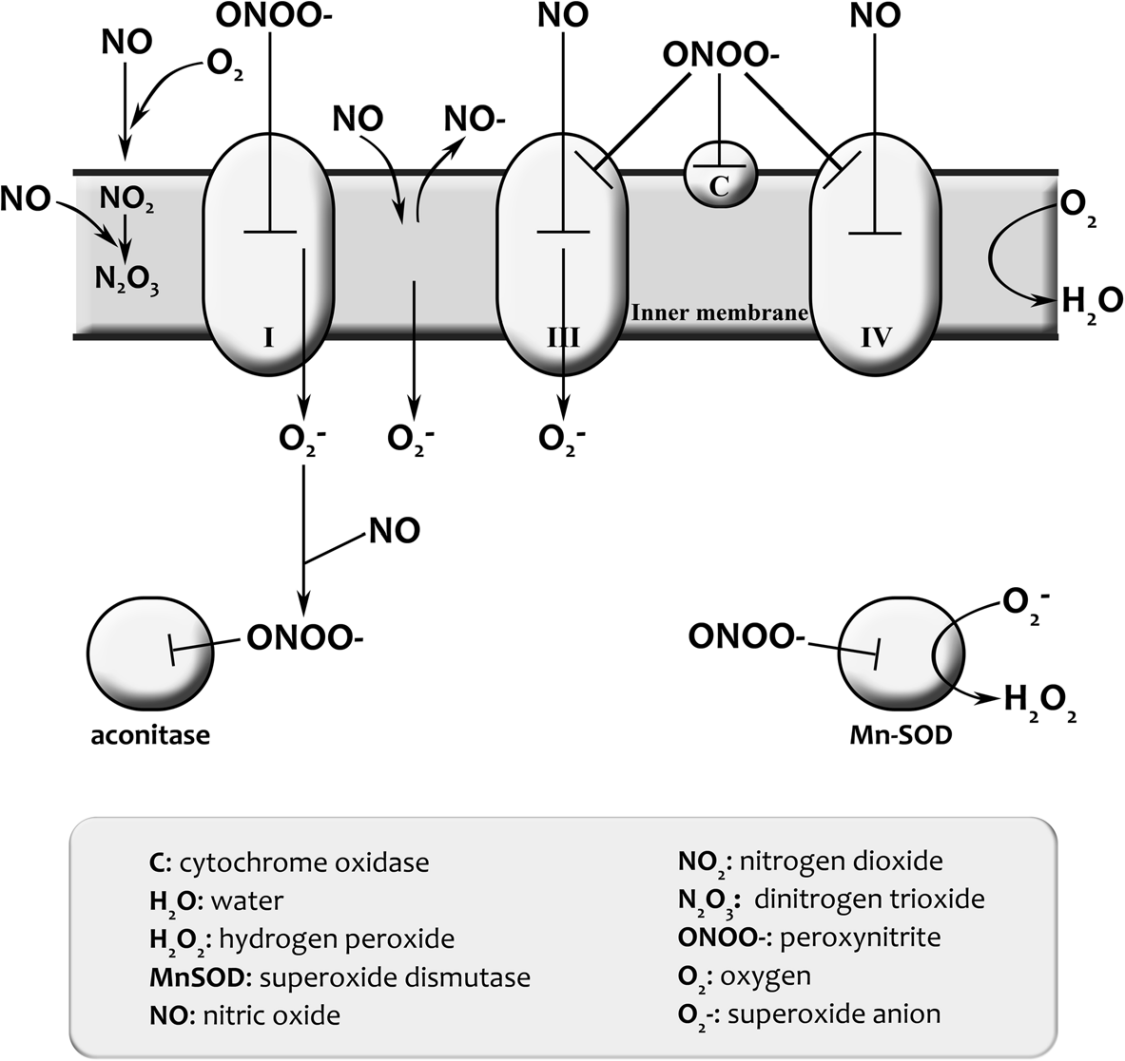
**Schematic representation of the inhibitory effects of NO and ONOO- on the ETC, enzymes of the tricarboxylic cycle and antioxidant enzymes.** NO and peroxynitrite inhibit the mitochondrial respiration via different mechanisms: NO itself causes selective, rapid, potent, but readily reversible inhibition of cytochrome oxidase and increased production of RNS within the intermembrane space. On the other hand, excessive levels of peroxynitrite and other RNS leads to slow, weak non-selective, but essentially irreversible inhibition of a wide range of mitochondrial components. Peroxynitrite inhibits Complex I, Complex II, cytochrome oxidase ATP synthase, MnSOD, aconitase, creatine kinase, and a plethora of other proteins playing an essential role in energy production. In addition, peroxynitrite is a potent oxidant capable of inducing peroxidation of mitochondrial membrane lipid components, hence increasing membrane permeability and disrupting the potential difference between the inner and outer membrane and inducing mitochondrial membrane transition. Inhibition of ATP production and electron chain dysfunction leads to the production of ever increasing production of ROS and RNS leading to a vicious circle culminating in eventual bioenergetic failure and often cellular necrosis or apoptosis.

**Figure 2:**
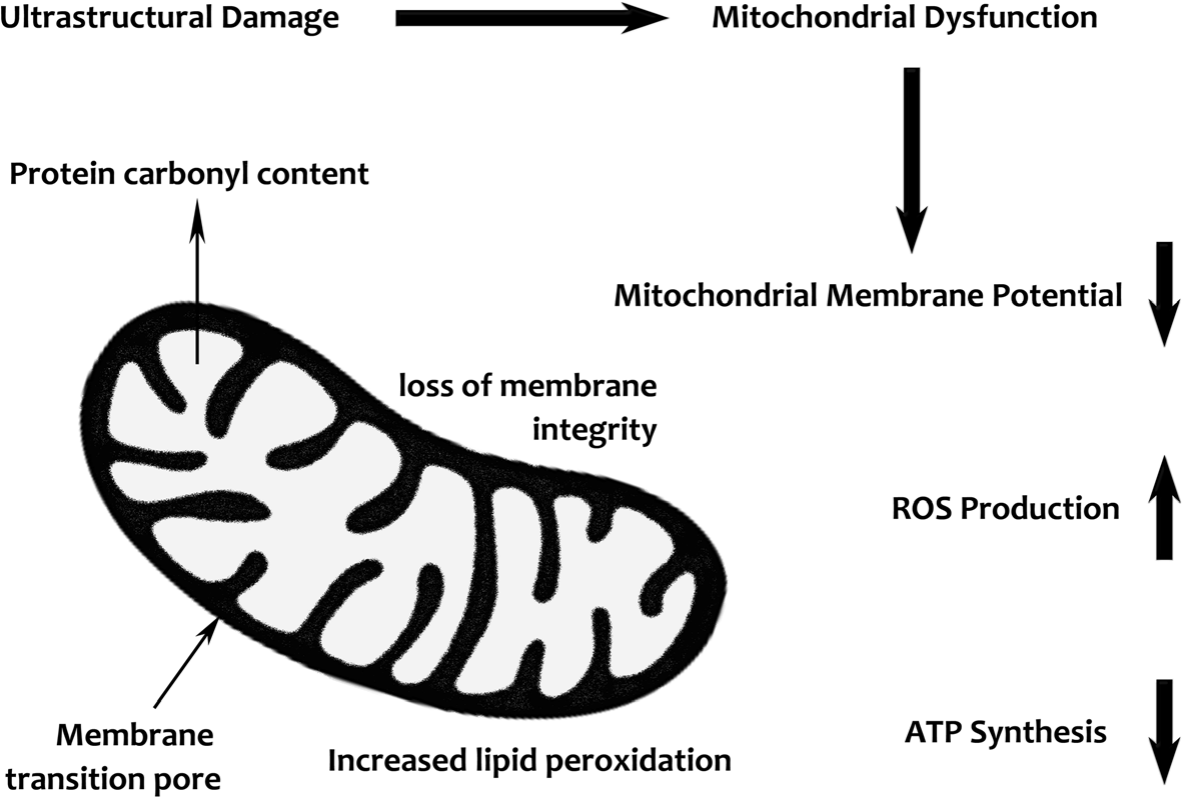
**Mitochondrial ultrastructural damage and impaired capacity for energy generation in an environment of chronic nitro-oxidative stress.** Excessive levels of peroxynitrite cause oxidative and peroxidative damage to lipids and proteins, leading to profound ultrastructural damage, including disrupted cristae, loss of outer membrane integrity, mitochondrial permeability transition, and uncoupling of ETC activity from oxidative phosphorylation.

Peroxynitrite can inhibit cellular energy production via yet another mechanism, the activation of poly [ADP-ribose] polymerase 1 [462,463], the chronic activation of which leads to impoverished levels of NAD+, an essential cofactor enabling the performance of the tricarboxylic acid cycle, glycolytic pathway, and the ETC [462,464-466]. Depletion of NAD+ thus results in severely diminished cellular ATP stores, resulting in profound cellular dysfunction [467,468]. Peroxinitrite can also grossly impair function of p53 by inducing conformational change in the transcription factor’s tertiary structure [469-471]. This altered structure impairs or even eliminates the capacity of the p53 protein to bind to DNA and thus exert its normal functions [472,469]. p53 plays a crucial role in coordinating increases in cellular metabolic activity to match increasing energy demands [473-475]. Loss of p53 facilitates the switch to anaerobic glycolysis as a source of ATP [476,477], resulting in dramatically reduced oxygen uptake and mitochondrial respiration [478] and a markedly diminished capacity for exercise [479]. Elevated levels of peroxynitrite can also impact the activity of proteins with a regulatory role in mitochondrial function, such as parkin and DJ-1, by inducing conformation changes leading to their loss of function or affecting post-translational signaling mechanisms rendering their protective actions ineffective [480]. Schematic representations of the deleterious effects of chronically elevated levels of ROS and RNS on mitochondrial function and energy production are presented in Figures 1 and 2 below.

### Pro-inflammatory cytokine (PIC)-mediated impairment of energy production

The adverse effects of elevated levels of TNF-α on mitochondrial biogenesis have already been discussed; however, excessive levels of TNF-α and other PICs, typifying a state of chronic systemic inflammation, can additionally disable oxidative phosphorylation [1]. This is evidenced by elevated levels of lactate levels and impaired mitochondrial function characteristic of chronic inflammatory states [481-483]. It is worthy of note, however, that this PIC-mediated suppression of ETC function is ultimately mediated by NO via a number of different mechanisms [484,485]. PICs can also inhibit mitochondrial respiration directly. TNF-α, one of the major PICs, can block electron transfer at Complex I [486,487], Complex III [488,489], and COX [490-492], leading to a significant reduction in the rate of respiration and the activities of the enzymes in the ETC [492,493]. TNF-α increases mitochondrial membrane permeability leading to membrane depolarization, increased intracellular calcium and a marked decrease in mitochondrial membrane potential [494,495], and increased generation of ROS [496,497]. TNF-α and IL-1β collude to inhibit the ETC and suppress pyruvate dehydrogenase activity [498,499]. IL-1β and TNF-α, acting in concert, have also been shown to increase aerobic glycolysis and inhibit oxidative phosphorylation [499]. Prolonged excessive levels of TNF-α also induces the development of aerobic glycolysis and appears to be yet another mechanism for inducing Warburg-like metabolism, whereby cells predominantly generate energy by glycolytic, non-oxidative breakdown of glucose, in an environment of excessive oxidative stress [490,491].

## Summary

This paper has detailed some of the evidence demonstrating the existence of immune dysfunction, oxidative stress, and mitochondrial dysfunction in many patients diagnosed with MS, PD, autism, bipolar disorder, depression, schizophrenia, and CFS. It is proposed that these apparently non-specific findings may contribute to the pathophysiology in each illness. Excessive levels of peroxynitrite, NO, and PICs clearly have the capacity to inhibit the activity of the ETC at several points, alone or synergistically, leading to the depletion of ATP production and promoting a switch to anaerobic glycolysis. Peroxynitrite and TNF-α can also depolarize the mitochondrial membrane via a number of different mechanisms once again having a deleterious effect on the generation of ATP. Peroxynitrite in particular can damage lipids and proteins, altering their conformation and function, causing structural damage to integral mitochondrial proteins and lipid membranes and to proteins regulating the function of the organelle. The capacity of peroxynitrite to inactivate a range of enzymes with an essential function in the generation of energy and the regulation of energy generation, such as p53, can provide other pathways to impaired oxidative metabolism. However, peroxynitrite is not alone in its ability to impair the activity of essential transcription factors as evidenced by the capacity of TNF-α to inhibit the production of PGC-1α and indirectly impair activity-stimulated mitochondrial biogenesis. It must be emphasized, however, that the presence of these inflammatory entities in an environment of oxidative stress is highly unlikely to be the sole cause of the mitochondrial dysfunction and impaired energy production seen in people with these illnesses. Genetic and epigenetic factors are also surely involved. Consequently, it is impossible to calculate the extent of the contribution that these entities make to the phenomenon of bioenergetic impairment seen in these apparently disparate illnesses, but it is likely that they play at least a part.

## Abbreviations

ATP: Adenosine triphosphate
BBB: Blood brain barrier
CAT: Catalase
CFS: Chronic fatigue syndrome
CNS: Central nervous system
COX: Cytochrome c oxidase
CSF: Cerebrospinal fluid
CWA: Children with autism
EDSS: Expanded disability status scale
ETC: Electron transport chain
GSH: Glutathione
IFN-γ: Interferon-γ
IL: Interleukin
iNOS: Inducible nitric oxide synthase
MDD: Major depression
MS: Multiple sclerosis
NO: Nitric oxide
PD: Parkinson disease
PGC-1α: Proliferator-activated receptor gamma coactivator-1
PIC: Pro-inflammatory cytokine
RNS: Reactive nitrogen species
ROS: Reactive oxygen species
SOD: Superoxide dismutase
TGF-β1: Transforming growth factor-β1
Th: T helper
TNF-α: Tumor necrosis factor-alpha
Treg: T regulatory
